# Inactivation of ${\text{H}}_{1}{\text{N}}_{1}$ viruses exposed to acidic ozone water

**DOI:** 10.1063/1.3242338

**Published:** 2009-10-29

**Authors:** Han S. Uhm, Kwang H. Lee, Baik L. Seong

**Affiliations:** 1 Department of Molecular Science and Technology, Ajou University, San 5 Wonchon-Dong, Youngtong-Gu, Suwon 443-749, Republic of Korea; 2 Department of Biotechnology, Yonsei University, 134 Shinchon-Dong, Seodaemun-Gu, Seoul 120-749, Republic of Korea

## Abstract

The inactivation of ${\text{H}}_{1}{\text{N}}_{1}$ viruses upon exposure to acidic ozone water was investigated using chicken allantoic fluids of different dilutions, pH values, and initial ozone concentrations. The inactivation effect of the acidic ozone water was found to be stronger than the inactivation effect of the ozone water combined with the degree of acidity, indicating a synergic effect of acidity on ozone decay in water. It is also shown that acidic ozone water with a pH value of 4 or less is very effective means of virus inactivation if provided in conjunction with an ozone concentration of 20 mg/l or higher.

The new flu known as swine flu, appearing almost daily in the news as of the summer of 2009, is a highly contagious disease. It has infected so many people worldwide in a short period of time, resulting in a high number of deaths.^1^ The World Health Organization declares influenza pandemic due to the high infection number of the new flu caused by the swine-original influenza $\text{A}\left({\text{H}}_{1}{\text{N}}_{1}\right)$ virus,^2^ one strain of ${\text{H}}_{1}{\text{N}}_{1}$ viruses. The best defense against this new flu will be the development of effective vaccines^1^ and medicines that can cure it. Authorities check people carefully in airports, train stations, and harbors, hoping to prevent the spread of the disease. They also decontaminate airplanes, trains, ships, and other transportation vehicles with disinfectants. The authorities also urge people to wash their hands and mouth with disinfectants to protect against this disease. There are a few new disinfectants.^3^ However, an environmentally friendly disinfectant is needed to fight these types of outbreaks. Acidic ozone water (AOW) is a very effective means of eliminating microbes. It is particularly useful for sterilizing bacterial endospores.^4^ In this context, the influence of AOW on the sterilization of ${\text{H}}_{1}{\text{N}}_{1}$ viruses (A/Singapore/6/86) is investigated here. AOW deactivates ${\text{H}}_{1}{\text{N}}_{1}$ viruses very effectively. After the disinfection of the viruses, AOW eventually becomes plain water^5–7^ and leaves no residue or harmful materials in the environment.

Acidic water is made from neutral water by mixing acidic materials into it. Mixing a small amount of acid such as hydrochloric acid (HCl) into water produces acidic water. The acidity of acidic water is represented by its pH value. 1 ton of acidic water with a pH value of 4 prepared from tap water may require 0.6 mol of hydrochloric acid, which is equivalent to 22 g of the acid. Clearly, a very small amount of acid is needed to create acidic water of pH 4 from tap water. An ozonizer from Ozone-Tech in Korea produced a high concentration of ozonized gas (approximately $120\;\text{g}/{\text{m}}^{3}$) after it was operated at 250 W with 1–2 lpm of oxygen gas in a scaled-down experiment in the laboratory of the authors. All of the experimental data presented below were obtained using AOW prepared from tap water for abundant availability in applications.

The ozone concentration n(t) in AOW decreases as time passes. The decay pattern of the ozone concentration in AOW may be a very complicated function of time, depending on the various contaminating components in the source water. Most of these contaminating components are likely to be organic materials that occur in nature. However, the ozone concentration is assumed to decay linearly according to

$$n\left(t\right)=n_{{\text{O}}_{3}}\;\text{exp}\left(-t/\tau \right),$$ (1)

which allows all subsequent calculations to be analytically tractable. Here, $n_{{\text{O}}_{3}}$ is the initial ozone concentration in acidic water and τ is the ozone decay time. However, the ozone decay profile in reality may be nonlinear due to several types of contaminating organic species.

The negative chlorine ions in AOW have a sterilizing effect on viruses. In fact, a strong acidity has a sterilizing effect^8–10^ on viruses in general. Assuming that N represents the microbe number in unit volume, the number of microbes killed per unit time and unit volume by AOW can be represented as^11^

$$\frac{dN}{dt}=-N\left\{\left[\alpha n_{{\text{O}}_{3}}\;\text{exp}\left(-\frac{t}{\tau }\right)\right]-\beta \right\},$$ (2)

where α is the inactivation coefficient of ozone in units of l/(mg min) and β is the kill rate of acidity in units ${\text{min}}^{-1}$. Integration of Eq. (2) over time t gives the density of the microorganisms in terms of time t,

$$\text{log}\left[\frac{N\left(t\right)}{N_{0}}\right]=-0.43\alpha n_{{\text{O}}_{3}}\tau \left[1-\text{exp}\left(-t/\tau \right)\right]-0.43\beta t,$$ (3)

where the constant $N_{0}$ represents the initial density of the microorganisms. An increase of the ozone decay time (τ) in Eq. (3) enhances the sterilization effect. Acidification of water increases the ozone decay time by several times compared to the decay time in neutral water,^12,13^ depending on various physical conditions. However, the ozone decay time decreases drastically when the water is contaminated by organic compounds.

The focus of this sterilization study is the inactivation of the influenza A virus-strain of ${\text{H}}_{1}{\text{N}}_{1}$, which is one type of human influenza virus. The virus inactivation in this study is loss of the reproducibility due to outside influences. Therefore, the following inactivation study concentrates mostly on loss of the infectivity. The evidence of inactivation is represented by the decrease of viable virus number estimated from virus titers^11^ after exposed to AOW. In order to observe the influence of organic compounds in nature on the ozone concentration and on the kill properties, an original virus suspension was prepared that consisted of a high concentration (100%) of chicken allantoic fluid, which is harmless to the virus. In fact, human influenza viruses tend to thrive in this type of prepared chicken allantoic fluid, which has high concentrations of many types of organic compounds. The survival data for ${\text{H}}_{1}{\text{N}}_{1}$ viruses exposed to AOW with pH values of 4 and 7 at the original high concentration (100%) of chicken allantoic fluid was observed. The ozone in AOW decays very quickly due to the high concentration of the chicken allantoic fluid in water. The high concentrations of organic materials from the chicken allantoic fluid hinder any inactivation mechanisms of the acidity and ozone on the viruses. Therefore, there is no evidence of any inactivation activity of AOW on the viruses at this high level of contamination with the chicken allantoic fluid, although there is a slight reduction in the number of viable viruses in AOW with a pH value of 4.

Dilution of the chicken allantoic fluid was done by mixing the chicken allantoic fluid with distilled water. For example, the solution contaminated with the virus at a dilution of 1/5 is made of 1 cc of chicken allantoic fluid contaminated with viruses mixed with 4 cc of distilled water. The virus inactivation experiment was then carried out by contact of one part of the virus-contaminated water to two parts of AOW. Inactivation of the viruses by acidic water of pH=4 exclusive of any ozone concentration was initially investigated for three different concentrations of the chicken allantoic fluid. Experimental data for the acidic water mixed with chicken allantoic fluids diluted by five times, ten times, and 25 times were obtained. The experimental data bunches together without depending sensitively on the dilution of the chicken allantoic fluid in the experimental range of 1/25–1/5. A straight line is obtained from $\text{log}\left(N/N_{0}\right)=-0.43\beta t$ for β=0.14/min least-squares fitted to the experimental data, which is consistent with Eq. (3), where N is the number of viable viruses remaining and $N_{0}$ is a control number. The degree of inactivation by the acidity at a pH value of 4 is marginal in comparison with the degree of inactivation by the ozone, as will be shown later.

Figure 1 shows plots of the log of the ratio of the number of viable viruses remaining (N) to the control number of $N_{0}$ versus the contact time t of the viruses to AOW mixed with the chicken allantoic fluid diluted by ten times for ozone concentrations of (a) 10 mg/l and (b) 20 mg/l. A separate virus-inactivation experiment was carried out for AOW at pH=3, resulting in sterilization of almost all of the viruses. Therefore, most of the subsequent presentations were restricted to AOW of pH=4 and 7. All of the experimental data henceforth in this article are the average values of three data samples. The error bars can be obtained from the square root of the second moment of data around its mean value. A typical error bar in Fig. 1(a) is shown at t=5 min at pH=4 and at an ozone concentration of 10 mg/l. An untouched control was analyzed each time to obtain the average control number $N_{0}=4.32\times 10^{5}$, which corresponds to $\text{log}\;N_{0}=5.6$. The curves in Fig. 1 are obtained from Eq. (3) for AOW at (a) $n_{03}=10\;\text{mg}/\text{l}$ and α=0.2 l/mg min, with τ=0.35 min for pH=7 and τ=1 min for pH=4; and at (b) $n_{03}=20\;\text{mg}/\text{l}$ and α=0.36 l/mg min, with τ=0.62 min for pH=7 and τ=1 min for pH=4. The inactivation coefficient α and the ozone decay time τ quoted here are tabulated in Table I and are least-squares fitted to the experimental data. The kill rate β in Eq. (3) of the acidity is assumed to be β=0.14/min, as mentioned earlier. The inactivation of viruses by AOW at the intermediate level of contamination by the chicken allantoic fluid is significant. The average control number $N_{0}=4.32\times 10^{5}$ corresponds to $\text{log}\;N_{0}=5.6$. Therefore, most of the viruses are killed by AOW at pH=4 in conjunction with an ozone concentration of 20 mg/l, as shown in Fig. 1(b), in which only 60 viruses out of $4.32\times 10^{5}$ survive at t=10 min.

Shown in Fig. 2 are plots of $\text{log}\left(N/N_{0}\right)$ versus the contact time t of the viruses to AOW mixed with the chicken allantoic fluid diluted by 25 times for an ozone concentration of 10 mg/L. The average control number $N_{0}=4.28\times 10^{5}$ corresponds to $\text{log}\;N_{0}=5.6$. In effect, all of the viruses in AOW at a pH value of 4 were killed at t=5 min, but one surviving virus at t=5 min out of ten inactivation attempts (i.e., N=0.1) was assumed for convenience regarding the log scale plot shown in Fig. 2. Therefore, the reference value of $\text{log}\left(N/N_{0}\right)$ for no virus was defined by $\text{log}\left(N/N_{0}\right)=-6.6$ (i.e., N=0.22). The curve in Fig. 2 is obtained from Eq. (3) for AOW at $n_{03}=10\;\text{mg}/\text{l}$ and α=0.5 l/mg min, with τ=1.2 min for pH=7 and τ=6.5 min for pH=4. Additionally, all of the viruses were inactivated by AOW at $n_{03}=20\;\text{mg}/\text{l}$ regardless of the pH value in this low level of contamination. In order to complete the inactivation study of viruses, the number of viable viruses remaining (N) after contact to AOW mixed with the high concentration of the chicken allantoic fluid diluted by five times for ozone concentrations was counted. The inactivation coefficient α and the ozone decay time τ obtained similarly to Figs. 1 and 2 for a high concentration of chicken fluid were also tabulated in Table I. The inactivation of viruses through AOW at an ozone concentration of 10 mg/l is negligible when tested in conjunction with the high concentration of the chicken allantoic fluid diluted by five times.

Several points are noteworthy in this virus-inactivation study. First, the ozone decay time τ must be independent of the initial ozone concentration in the linear ozone decay represented by Eq. (1). However, the ozone decay time at $n_{03}=20\;\text{mg}/\text{l}$ regardless of the pH value is considerably longer than that at $n_{03}=10\;\text{mg}/\text{l}$ in Table I, indicating nonlinearity of the ozone decay data. This nonlinearity may be caused by multiple components of the organic materials in the chicken allantoic fluid. The ozone may decay very quickly at the beginning due to the organic material from the chicken allantoic fluid, but it may decay slowly at a later stage after the organic materials are gone. This stepwise disappearance of the organic materials may also cause the nonlinearity in the ozone decay data. Second, the pH value of 4 in Figs. 1 and 2, and in Table I represent its value in the AOW. However, in reality, two parts of AOW were mixed with one part of contaminated water at pH=7 in the inactivation experiment. Therefore, the amount of hydrochloric acid for two parts of the acidic water at pH=4 was used to remake the acidic water of three parts in volume, resulting in a pH value of 5.3, as indicated in Ref. 4. It is also shown in Ref. 4 that two parts of acidic water at pH=3 mix with one part of water at pH=7, resulting in three parts of acidic water at pH=4. In fact, a virus-inactivation experiment with AOW at pH=3 was conducted in our experiment, resulting in the inactivation of virtually all of the viruses. There is no contaminated water at pH=7 in nature for the dilution of the acidity when AOW is sprayed for virus inactivation. Therefore, the acidity of AOW sprayed in the dry environment preserves its original value, thereby prolonging the ozone decay time and effectively inactivating viruses. In this context, virus inactivation by AOW may be more pronounced in nature than in the present experiment. Third, a TEM image^14^ of *E. coli* cells treated by AOW at pH 4 and with ozone concentration of 20 mg/l indicates the destruction of cell envelopes, showing nearly empty cells. Therefore, it is believed that AOW may also destroy virus envelops, inactivating their infectivity.

Table I summarizes the results of this inactivation study of ${\text{H}}_{1}{\text{N}}_{1}$ viruses in which AOW was used. The ozone decay time τ in AOW with a pH value of 4 is always longer than that when the pH value is 7, clearly indicating the synergic effect^12^ of acidic water on the ozone decay activities in water. Therefore, the inactivation effect of AOW is stronger than the sum of the inactivation effect of the ozone water and that of the acidity. “Not applicable” (N/A) in Table I indicates that all of the viruses were inactivated. Hence, experimental measurements are not available, and data could not be entered into the table. The scientific conditions corresponding to a number less than −3 or N/A in the right column [$\text{log}\left(N/N_{0}\right)$ at 10 min] in Table I are suitable for virus inactivation. In this context, it is concluded that AOW at a pH value of 4 or less is one of the most effective means of virus inactivation given an ozone concentration of 20 mg/l or higher.

## Figures and Tables

**Table I.** The inactivation coefficient α, the ozone decay time τ, and the log reduction at t=10 min for AOW with several different dilutions of chicken allantoic fluid at pH values of 4 and 7.

Dilution	$n_{\text{O}3}$ (mg/L)	α (l/mg min)	pH	τ (min)	$\text{log}\left(N/N_{0}\right)$ at t=10 min
1/5	10	0.2	7	0.1	−0.08
			4	0.3	−0.69
	20	0.24	7	0.43	−1.06
			4	0.55	−1.66
1/10	10	0.2	7	0.35	−0.37
			4	1	−1.54
	20	0.36	7	0.62	−2.13
			4	1	−3.86
1/25	10	0.5	7	1.2	−2.8
			4	6.5	N/A
	20	N/A	7	N/A	N/A
			4	N/A	N/A

**FIG. 1.**
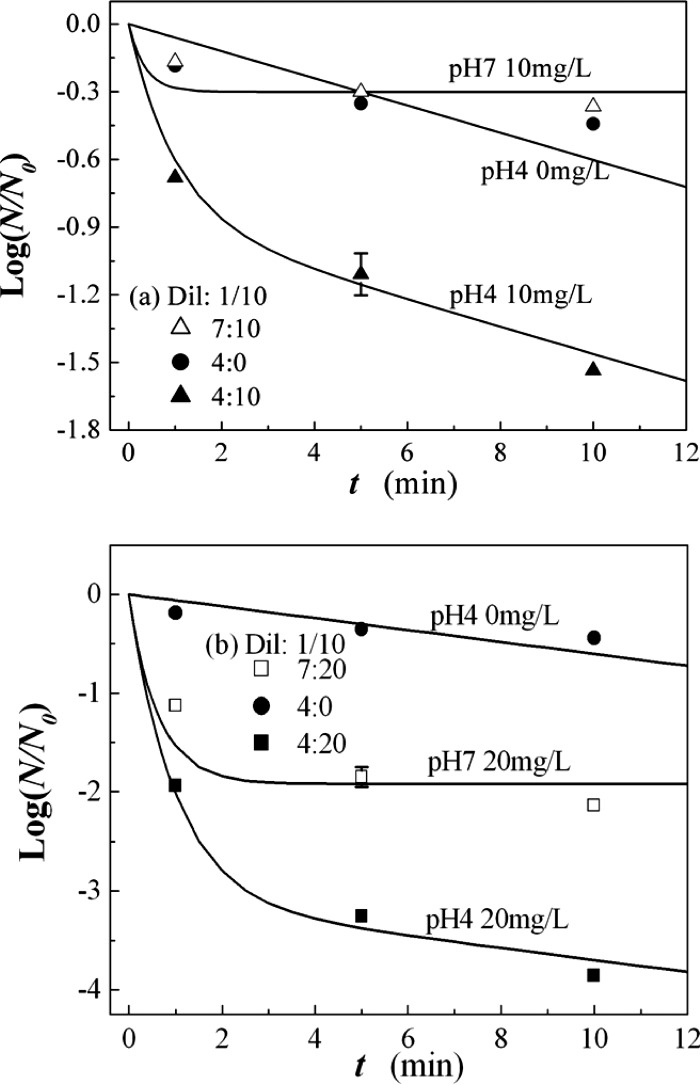
Plots of $\text{log}\left(N/N_{0}\right)$ vs the contact time t of the viruses to AOW mixed with the chicken allantoic fluid diluted by ten times for ozone concentrations of (a) 10 mg/L and (b) 20 mg/L. The curves are obtained from Eq. (3) for AOW at (a) $n_{{\text{O}}_{3}}=10\;\text{mg}/\text{l}$ and α=0.2 l/mg min, with τ=0.35 min for pH=7 and τ=1 min for pH=4; and at (b) $n_{{\text{O}}_{3}}=20\;\text{mg}/\text{l}$ and α=0.36 l/mg min, with τ=0.62 min for pH=7 and τ=1 min for pH=4.

**FIG. 2.**
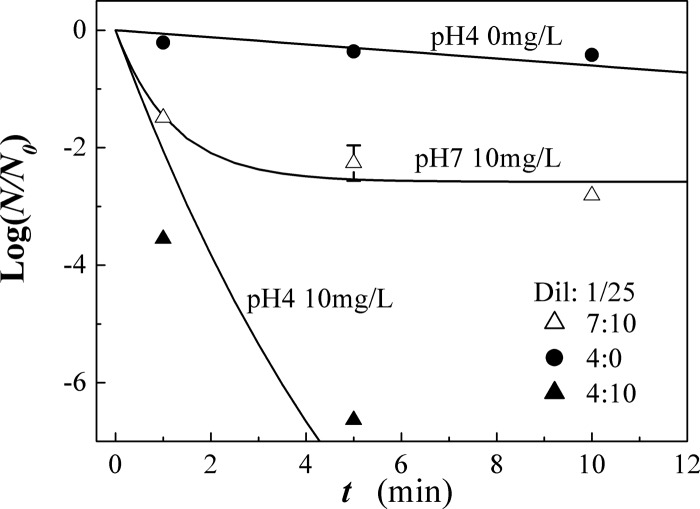
Plots of $\text{log}\left(N/N_{0}\right)$ vs the contact time t of the viruses to AOW mixed with the chicken allantoic fluid diluted by 25 times for an ozone concentration of 10 mg/l. The curve is obtained from Eq. (3) for AOW at $n_{{\text{O}}_{3}}=10\;\text{mg}/\text{l}$ and α=0.5 l/mg min, with τ=1.2 min for pH=7 and τ=6.5 min for pH=4.
